# Giardiasis as a neglected disease in Brazil: Systematic review of 20 years of publications

**DOI:** 10.1371/journal.pntd.0006005

**Published:** 2017-10-24

**Authors:** Camila Henriques Coelho, Maurício Durigan, Diego Averaldo Guiguet Leal, Adriano de Bernardi Schneider, Regina Maura Bueno Franco, Steven M. Singer

**Affiliations:** 1 Biology Department, Georgetown University, Washington DC, United States of America; 2 U.S. FDA, OARSA, CFSAN, Laurel, MD, United States of America; 3 Basic Pathology Department, Biological Sciences Sector, Federal University of Paraná, Curitiba, Brazil; 4 Department of Bioinformatics and Genomics, University of North Carolina at Charlotte, Charlotte, NC, United States of America; 5 Animal Biology Department, Biology Institute, University of Campinas, Campinas, Brazil; Universidade Federal de Minas Gerais, BRAZIL

## Abstract

**Introduction:**

Giardiasis is an intestinal infection that affects more than two hundred million people annually worldwide; it is caused by the flagellated protozoan *Giardia duodenalis*. In tropical countries and in low or middle-income settings, like Brazil, its prevalence can be high. There is currently no systematic review on the presence of *G*. *duodenalis* in patients, animals or water sources in Brazil.

**Methods:**

This systematic review was performed according to recommendations established by Preferred Reporting Items for Systematic Reviews and Meta-Analysis (PRISMA). As databases for our searches, we have used PubMed, Embase, Scopus and the Brazilian database SciELO using the keywords Giardia^*^ and Brazil.

**Results:**

This systematic review identified research studies related to *G*. *duodenalis* in water, giardiasis in animals, prevalence of giardiasis across Brazilian regions, genotyping of strains isolated in humans, and giardiasis in indigenous populations. We also propose a network of *G*. *duodenalis* transmission in Brazil based on genotypes analyses.

**Conclusion:**

This is the first time within the last twenty years that a review is being published on the occurrence of *G*. *duodenalis* in Brazil, addressing relevant issues such as prevalence, molecular epidemiology and analytical methods for parasite detection.

## Author summary

Giardiasis is an intestinal disease that affect millions of people worldwide, including children. Its main route of transmission is by ingestion of food or water contaminated with the protozoan *G*. *duodenalis*. Transmission does not require an animal host, although transmission from animals to human (zoonotic transmission) has been confirmed as an important vector of human giardiasis. This study is a comprehensive description of the impact of giardiasis in Brazil based on studies published in the country from the past 20 years. We describe *Giardia* prevalence in humans (including indigenous populations), animals and water supplies. In addition, we create a transmission network model for the disease, based on genotype data previously identified in animal and human hosts as well as in environmental samples. The data compiled here will be useful for design of policies to prevent giardiasis transmission in Brazil.

## Introduction

*Giardia duodenalis* is a non-invasive protozoan that attaches to the mucosa of the small intestine in infected hosts leading to giardiasis—a disease that is characterized by a range of clinical symptoms (mild, moderate, or severe) or even asymptomatic infection in many cases. Giardiasis affects more than 280 million people annually worldwide [1] and its transmission occurs by the ingestion of cysts through contaminated water and food or through person-to-person contact (i.e., fecal-oral transmission). In low and middle-income countries, the prevalence of giardiasis can reach up to 30%. In many cases, lower socio-economic status is associated with a higher prevalence of the disease as there is a greater risk of exposure to contaminated water in poor communities [2, 3]. Although the epidemiology regarding transmission is highly variable [4] and remains a contentious issue, giardiasis can be considered a zoonotic disease. [5, 6].

Eight genetic groups (or assemblages) of *G*. *duodenalis* (A to H) have been identified: assemblages A and B are considered zoonotic, infecting both humans and animals, including domestic animals, rodents and livestock. Other assemblages infect many species of animals. For example, assemblages C and D are usually infective to dogs and assemblage E is often found in ruminants [6]. Recent studies have shown that assemblages C and E are also able to infect humans [7, 8], although this seems to be a rare occurrence.

Giardiasis is a disease closely related to low-income and lack of sanitation infrastructure [9]. Although Brazil has improved in infrastructure and educational levels in recent years, the country still presents a disparity among its regions. While in the Southwest region 82.3% of the houses have adequate sanitation systems, in the Northern region this coverage is only 22.4%. Much of this discrepancy is due to disparities in the sanitation services among the different social strata [10]. Brazil is a very large country (8,515,767,049 km^2^) and currently has a population of more than 190 million people [10].

In this study, the first systematic review on giardiasis in Brazil, we evaluated the studies published from 1995 to 2015 that address giardiasis as a concern for public health in the country. We describe prevalence of giardiasis across the states, the most frequent assemblages found in humans and animals, the geographic location and distribution of different parasitic assemblages, and contaminated water as a source of *Giardia* cysts.

## Methods

### Systematic review

This systematic review was performed according to recommendations established by Preferred Reporting Items for Systematic and Meta-Analysis (PRISMA) [11], a statement of items for reporting systematic reviews. The authors searched in the U.S. National Institutes of Health's National Library (PubMed), Scopus, Embase and in the Brazilian database SciELO using the keywords «Giardia^*^» and «Brazil». This systematic review identified research studies related to *Giardia* in water, giardiasis in animals, prevalence of giardiasis across Brazilian regions, genotyping of strains isolated in humans and giardiasis in indigenous populations.

### Search criteria

Searches for each topic were restricted to studies published between January 1995 and December 2015, in English, Portuguese, French or Spanish. Data were abstracted from each of the selected articles independently, using a standardized Excel sheet for the sub-themes: a) Detection of *Giardia* cysts in water samples in Brazil, b) Detection of *Giardia* in pets, farm animals and wild animals in Brazil, c) Prevalence of giardiasis in the Brazilian population across the states, d) Giardiasis in the Brazilian indigenous population, and e) Distribution of *Giardia* assemblages in human hosts across Brazil.

Articles that either did not contain relevant information (a-e) or contained only information related to laboratory analysis such as morphological, molecular and biochemical analysis of *Giardia* were excluded. Articles without full text access were also excluded after attempts to search in other databases and direct contact with corresponding authors. The last date searched was November 1^st^, 2016.

**S1 Fig** describes the procedure used to obtain the articles used in this study according to PRISMA. **Table 1**shows the number of articles from each database included in the analyses. A map with Brazilian territory and its population density was constructed using the ArcGIS software.

**Table 1:** Result of each database search for the terms «Giardia^*^» and «Brazil». Searches for articles in Portuguese, English, Spanish and French were made using four databases, including the Brazilian SciELO.

Database	Hits	After Exclusion and Duplicates
**PubMed**	*334*	*125*
**Embase**	*272*	*35*
**Scopus**	*309*	*39*
**SciELO**	*134*	*25*
**TOTAL**	***1*,*049***	***224***

### Transmission networks analyses

Based on the literature review of giardiasis studies that were performed in Brazil from 1995 to 2015, we extracted gene sequences deposited in Genbank from all the studies related to humans, animals and water, that were isolated in Brazil. Isolate sequences were extracted from Genbank on National Center for Biotechnology Information (NCBI; ncbi.nlm.nih.gov) nucleotide database.

A total of 633 sequences were identified matching the Brazilian isolates for three genes available for *G*. *duodenalis*: beta-giardin (*bg)*, glutamate dehydrogenase (*gdh*) and triose phosphate isomerase (*tpi*) **(Table 2)**.

**Table 2:** Brazilian isolates sequences available on NCBI for *bg*, *gdh* and *tpi* genes. Isolate sequences were extracted from Genbank or NCBI database.

Gene	Isolates	Isolation Sources^1^	Categories^2^
*Beta-giardin (bg)*	*143*	*5*	*5*
*Glutamate dehydrogenase (gdh)*	*343*	*22*	*8*
*Triose phosphate isomerase (tpi)*	*147*	*15*	*6*

1- Isolation Sources = Original hosts before standardized into categories.

2- Categories: Non-Human primates, Farm animals, Dogs, Cats, Humans, Environmental Samples, Wildlife and Animals

Multiple sequence analyses were performed on *bg*, *gdh* and *tpi* genes. For this analysis, two *gdh* sequences that were associated with vegetables rather than water, animals or humans were excluded (KJ741292 e KJ741293). A search for duplicate sequences was performed but no sequences were excluded to avoid loss of host data due to the presence of sequences with 100% identity in multiple hosts.

For phylogenetic analysis, a *bg* gene from *Giardia cati* (KP798445.1), a *gdh* gene from *Giardia psittaci* (AB714978.1) and a *tpi* gene from *Giardia microti* (AY228649.1) were selected as outgroups. Nucleotide sequence data for 144 *bg*, 342 *gdh* and 148 *tpi* genes were aligned separately using MAFFT v.7.215 [12] under default settings. The alignments were visualized in Mesquite v.3.04 [13].

Phylogenetic analyses were performed on the three alignments using maximum likelihood as implemented in RAxML [14]. Host data was manually extracted from the original papers and categorized into seven different categories: Non-Human primates, Farm animals, Dogs, Cats, Humans, Environmental Samples and Wildlife Animals. Host data was associated with tree data utilizing a character matrix and data was mapped onto the phylogenies **(S3, S4 and S5 Figs)**.

Utilizing a similar approach as Janies [15] we used betweenness centrality to calculate the connectedness of hosts. To generate a transmission network, an apomorphy list (changes of host) was extracted from each phylogenetic tree. The apomorphy list holds the information of the shift from one host to another based on the relationships of ancestry between the different sequences, calculated on the phylogenetic trees, and the metadata (host) associated with it, thus, giving directionality to the graph. The graph generated is based on direction and frequency of transmission between the hosts observed on the phylogenetic tree and the relative size of each node is based on the Centrality Score of the given node within the network.

## Results

### Detection of *Giardia* cysts in water samples in Brazil

The analyses of pathogenic protozoa in water samples in Brazil was initiated in the early 2000s. However, there have been no well-documented waterborne giardiasis outbreaks in the country through the period covered by this study.

Contamination with *G*. *duodenalis* cysts was first documented in surface water samples from Brazil in 2001 by Franco et al [16]. Sampling was performed in the Atibaia River, in the city of Campinas, Southeastern Brazil during three consecutive weeks. The membrane filtration technique was employed to concentrate cysts using acetate cellulose membranes of 47 mm diameter and 3 μm porosity. For the recovery of this parasite, two different procedures were compared: either rinsing and scraping the membrane surface (RM method) or dissolving the membrane in acetone (ADM method). Cysts were visualized by a direct immunofluorescence assay (IFA) (Merifluor kit, Meridian Diagnostic, Cincinnati, Ohio). All water samples were positive for *Giardia*, despite the high turbidity. The RM method showed higher recovery rates in positive control assays.

Although some studies [17–19] utilized the USEPA reference method 1623 (concentration by IDDEX FiltaMax followed by purification with Immunomagnetic Separation (IMS) and immunofluorescence assay (IFA) [20] or the calcium carbonate flocculation technique for concentration of *Giardia* cysts in different sources of water [17, 19, 21, 22], membrane filtration followed by immunofluorescence assay remains the most employed technique in the country for the detection of this parasite **(Table 3)**. Sampling volumes ranged from 0.5 L to 1000 L. The USEPA Method 1623 was employed in only a few studies [17–19], probably due to its excessive cost being prohibitive for most Brazilian laboratories, except for those located in the Southeast region of the country.

**Table 3:** Detection of *G*. *duodenalis* cysts in water sources derived through different regions of Brazil. Only studies using the membrane filtration technique and immunofluorescence or molecular assays are included.

Water Source	City (State)	Filtered Volume	Number of Samples	Frequency of Positivity	Range of Number of cysts/L	Reference
Surface water	Campinas (São Paulo)	0.5L	03	100.0%	33.0–95.0	[16]
Surface water	Campinas (São Paulo)	1L	08	87.5%	2.5–120^+^	[24]
Surface water	Porto Said and Santa Maria da Serra	5L	28	0	A^+^	[34]
Surface water; treated water	Maringá (Paraná)	1,000L	15 (surface water); 15 (treated water)	19.9% (surface water); A (treated water)	0.026–0.2	[26]
Surface water; treated water	Londrina (Paraná)	30L (raw water); 100L (treated water)	24 (surface water); 24 (treated water)	8.3% (raw water); 0 (treated water)	0.42–4.2	[25]
Surface water; spring water	Piracicaba, São Lourenço da Serra, São Paulo (São Paulo)	10L	11 (surface water); 1 (spring water)	36.3% (surface water); 0 (spring)	NA	[30]
Spring water	Campos do Jordão (São Paulo)	20L	72	2.7%	0.07–0.1	[35]
Brackish/estuarine water	Cananéia (São Paulo)	1L – 10L	44	18.1% - 36.3% (according to sampling site)	NA^+^	[32]
Bottled mineral water*	Campinas (São Paulo)	1.5L – 2.0L	26	0	A	[36]
Seawater	Florianópolis (Santa Catarina)	1L – 10L	04	25.0%	NA^+^	[33]
Groundwater (rural area)	Maringá (Paraná)	100L	40 (from artesian wells; 40 (from commons wells); 01 (of mine)	0	A	[37]

* = nongaseous mineral waters

**A** = Absence on IFA (immunofluorescence assay)

**NA** = not applicable; positive by PCR (Polymerase Chain Reaction)

^**+**^ = Samples purified by ImmunoMagnetic Separation (IMS)

It is relevant to emphasize that surface water is more commonly used for the supply of drinking water than underground or spring water sources in Brazil. Taken together, the data from Table 3, allied with other studies [17, 19, 22] denote the wide occurrence of *G*. *duodenalis* in surface waters in Brazil, and highlight the necessity for water treatment companies to comply with Ordinance 2914/2011 [23] to ensure a safe water supply for Brazilian population and to minimize public health risks.

Moreover, few studies have shown recovery efficiency data: when Method 1623 was the chosen methodology, recoveries ranged from 34% to 39.4% [17–19] in accordance with USEPA’S recommendation. Matrix spiked sample assays conducted by membrane filtration and calcium carbonate flocculation, without or with IMS, showed an increase in recovery efficiencies when the IMS step was added to both methodologies [24].

Few studies have addressed the prevalence of *G*. *duodenalis* cysts in treated waters [17, 25, 26]. In the metropolitan region of São Paulo state, which has undergone accelerated population growth, *G*. *duodenalis* cysts were detected in treated water produced by a conventional water treatment system [17]. The detection of the parasite in water samples was performed using the USEPA Method 1623. *Giardia* cysts were detected in 41.7% of treated water samples, with concentration ranging from non-detected to 0.06 cysts/L. In other studies, all treated water samples were negative [25, 26].

In the past, most studies concerning G. *duodenalis* in water were performed in states of the Southeastern and Southern regions of Brazil. Very few studies were conducted in other regions, likely reflecting the social disparities existing throughout the country. Two studies [27, 28] were conducted in Pernambuco (a state in the Northeast region). The first study found a prevalence of 50.0% for *Giardia* cysts in samples from the Beberibe River which suffers anthropogenic and animal contamination as demonstrated by high levels of *E*. *coli* found in its water (from 50,000 to ≥ 160,000 NMP/100mL) [27]. The second study investigated bacterial and parasite contamination of rainwater stored in tanks and clay pots, in a semi-arid region of Northeast Brazil. G. *duodenalis* cysts were detected in 10.0% of rainwater samples conserved in clay pots and tanks, respectively [28]. In both studies, traditional methodologies such as spontaneous sedimentation and light microscopy were employed, and cysts were visualized on slides by Lugol staining.

In Brazil, the presence of thermotolerant coliforms is still often used to verify water potability, and when combined with other water characteristics such as turbidity, it is an indirect indicator for the presence of potentially pathogenic protozoa [29]. In most studies, no correlation was found between the occurrence of G. *duodenalis* in raw water samples and bacteriological, physical, chemical or climate factors. However, in the Paraná state, a correlation was found between the presence of G. *duodenalis* and high mean value of turbidity– 1,198.85 NTU (r = 0.5809; p = 0.0029) [25].

The accurate detection of this protozoan and its molecular characterization is still incipient or non-existent in many parts of the country. To address the probability of G. *duodenalis* infection from water wells in a peri-urban area, genotypic characterization of the protozoan was performed. Sequence analysis from the *gdh* gene yielded predominantly genotype A–subgenotype II [18].

Additional genotypic characterization of G. *duodenalis* contaminating surface waters was conducted in two municipalities within the São Paulo state. Phylogenetic analyses based on *gdh* gene sequences also showed the predominance of *G*. *duodenalis* belonging to genotype AII, the most common genotype associated to human giardiasis in this region [30].

Evaluation of the genetic diversity of a set of environmental water samples (river and stream waters) from the metropolitan region of Campinas, São Paulo, found that most of the samples contained assemblages A and B. In addition, assemblages C and D were also identified at the water abstraction point of the city. Only one sample (from the Anhumas River), presented mixed assemblages (BIV and D) determined through amplification and sequencing reactions using the *gdh* and *tpi* genes [31].

Contamination by G. *duodenalis* cysts was monitored in an important mariculture production area in São Paulo state. The search for the protozoan was performed in brackish waters in all stages of oyster cultivation and treatment steps before to be sent to market. In addition, analyses included search for *Giardia* in a recreational area frequented by tourists. PCR amplification of the *bg* gene demonstrated contamination by *G*. *duodenalis* in all analyzed sites [32].

In Florianópolis, Santa Catarina state, the evaluation of tropical water sources in the main shellfish growing areas, revealed contamination by *G*. *duodenalis* (assemblage A) in one site of seawater highly impacted by domestic sewage [33].

Few studies have addressed the issue of microbiological risk for *Giardia*. A Quantitative Microbial Risk Assessment (QMRA) based on parasite concentration to estimate the probability of protozoan parasite infection associated with water ingestion was conducted in four densely urbanized regions of São Paulo state [19]. The estimated risk of *Giardia* infection ranged from 0.29% to 2.47% per year for adults, and from 0.08% to 0.70% for children. The infection risk by this parasite was higher than what is considerable tolerable by the USEPA for a yearly exposure. G. *duodenalis* risk infection was greater for adults than that observed for children, reflecting the higher water ingestion in adults compared to children. The study also concluded that the metropolitan region of Campinas exhibits the highest risk of G. *duodenalis* infection among all studied regions.

### Detection of *Giardia* in pets, farm animals and wild animals in Brazil

**Fig 1:**
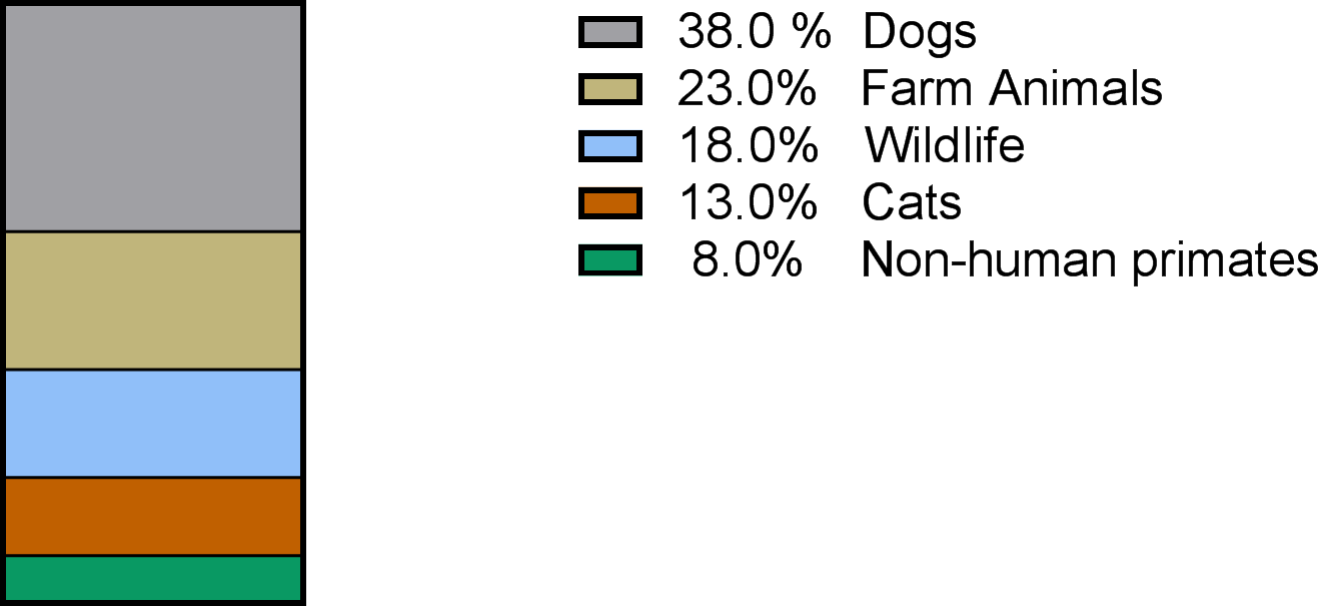
Proportion of studies of *G*. *duodenalis* in Brazil performed in different animal hosts.

Although G. *duodenalis* is an important cause of gastrointestinal disease in humans, it has also been frequently diagnosed in wildlife and companion animals. The detection of this protozoan in animals has been widely documented in numerous hosts, including dogs, cats, calves, sheep, lambs, horses, pigs, non-human primates, and wildlife, in many regions of Brazil. Among the 61 studies that reported *G*. *duodenalis* cysts in these hosts between 1995 and 2015 (Fig 1), dogs were the most studied host (38%) followed by farm animals (23%) and wildlife (18%).

**Fig 2:**
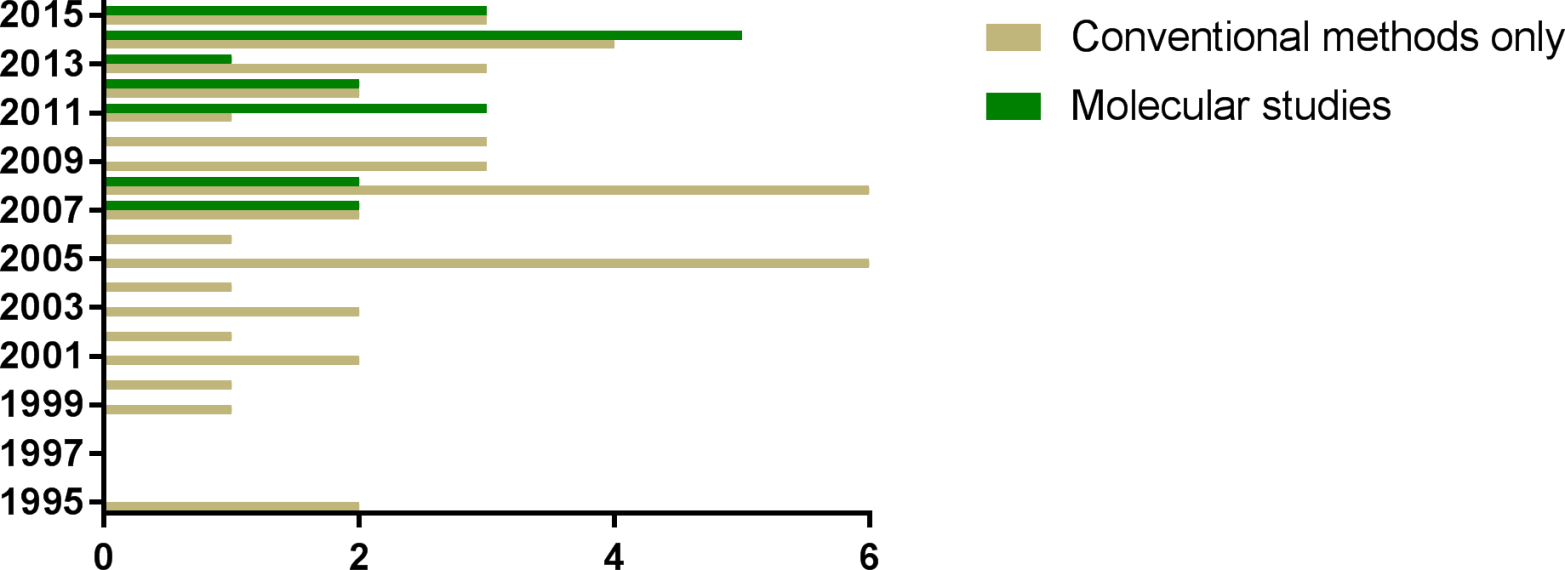
Number of studies in which conventional and molecular methods were used for diagnosis of giardiasis in animals between 1995 and 2015.

Conventional diagnosis based on optical microscopy was the only method in 68.8% (42/61) of the studies. The development of molecular markers has allowed the identification of specific assemblages in both animal hosts and human patients; the first studies with DNA-based approaches for assessing *Giardia* infection in animals were only published in 2007 [38, 39]. Examination of the last five years covered by this literature review, showed that molecular techniques such as Polymerase Chain Reaction (PCR) and DNA sequencing have been used in 53.8% (14/26) of the included studies and only studies published in the last two years of our analysis presented greater numbers of molecular diagnoses compared to techniques based on optical microscopy **(Fig 2)**.

The Brazilian canine population is estimated at 28 million, including over 22 million stray dogs [40], which can be explained by the great availability of food in the streets (obtained from garbage) and the climate conditions [41–43]. The prevalence of *Giardia* cysts in dogs in Brazil ranged from 0.8% [44] to 45% [45]. In many regions of Brazil, this prevalence is between 8.4% to 11.1% based on microscopic examinations [46]. The genetic characterization studies using DNA-based approaches detected mainly host adapted genetic assemblages C and D, even though the assemblages AI, AII, BIII and BIV have also been reported [31, 34, 39, 47, 48]. Thus, zoonotic transmission could represent a public health problem in developing countries [41]. When compared to other countries, there is still relatively little information on *G*. *duodenalis* assemblages in dogs [49] even though the predominance of host adapted assemblages C and D is notable.

Reports of assemblages A and B in dogs suggest that zoonotic transmission could represent a problem of public health in Brazil [41]. Both domestic and stray animals can be disseminators of zoonotic parasites. In Brazil, the prevalence of *G*. *duodenalis* in stray dogs is higher in comparison to household pets [41, 45, 50]. A statistically significant difference was also found between shelter dogs and household pets, probably due to the greater concentration of animals and exposure to environmental contamination [45], which can also be considered for stray dogs. A consistent program of sanitary education must be included in public health actions for the control of intestinal parasites in dogs [41].

Cats may also represent an important reservoir of *G*. *duodenalis* based on prevalence and the genotypes that have been identified in Brazilian studies. The prevalence ranges from 3.5% to 13.7% [51, 52] and most molecular studies detected the potential zoonotic genetic assemblages AI, BIII and BIV [31, 38, 39]. Wild felines may also represent a significant source of infection by *G*. *duodenalis*. The prevalence can reach 38.5% in captive felines with 23.1% having mixed infection with helminths [42, 53].

The presence of *Giardia* is also commonly reported in livestock, although most studies are restricted to cattle. These studies report that calves usually shed *G*. *duodenalis* cysts from the host adapted assemblage E. However the zoonotic assemblages A and B have also been identified [31, 54, 55]. Thus infected calves may also represent a public health risk [55]. The presence of *G*. *duodenalis* is rarely analyzed in goats, but in two recent studies in Brazil, prevalence of the parasite ranged between 22.6% and 29.3% and the dominant genotype was genetic assemblage E [56, 57]. A similar scenario is observed for studies with sheep. The prevalence of *G*. *duodenalis* in sheep ranged from 24% to 34% and the same host adapted assemblage was detected [54, 58]. Regarding equines, low prevalence was detected and no molecular studies have been published [59, 60].

The presence of cysts of *G*. *duodenalis* in wildlife animals has been evaluated by many studies in Brazil, most of them based on conventional diagnostic techniques. A single study with chinchillas, ostriches and a jaguar detected genetic assemblages AI and B [61]. In small wild rodents, a prevalence between 2.05 and 100% was reported. However, apart from the single exception just mentioned, there are no data regarding the genetic assemblages in wildlife [62–67]. A prevalence of 3.6% was found in captive snakes based on enzyme immunoassay [68].

In non-human primates, several studies have reported a prevalence between 0.5% and 44.4% [69–72]. The genetic assemblage A was detected in primates kept in a zoo, which highlights that, considering the zoonotic potential of the assemblage detected, regular coproparasitological surveys are necessary to safeguard the captive animals, their caretakers and people visiting zoos [69]. The genotype AI was also detected in captive *Alouatta clamitans* in the south of the country, indicating that these animals might be susceptible to infection with *G*. *duodenalis* strains of human origin [72, 73].

The states from the southeast region of Brazil are responsible for 52% (São Paulo 32%, Rio de Janeiro 15%, Minas Gerais 13% and Espírito Santo 2%) of the publications examined. In contrast, the North, Northeast and Middle West regions together are responsible for only 13.3% of all studies, which represents another reason for the inequality between regions.

### *G*. *duodenalis* and its assemblages in humans across Brazilian states

**Fig 3:**
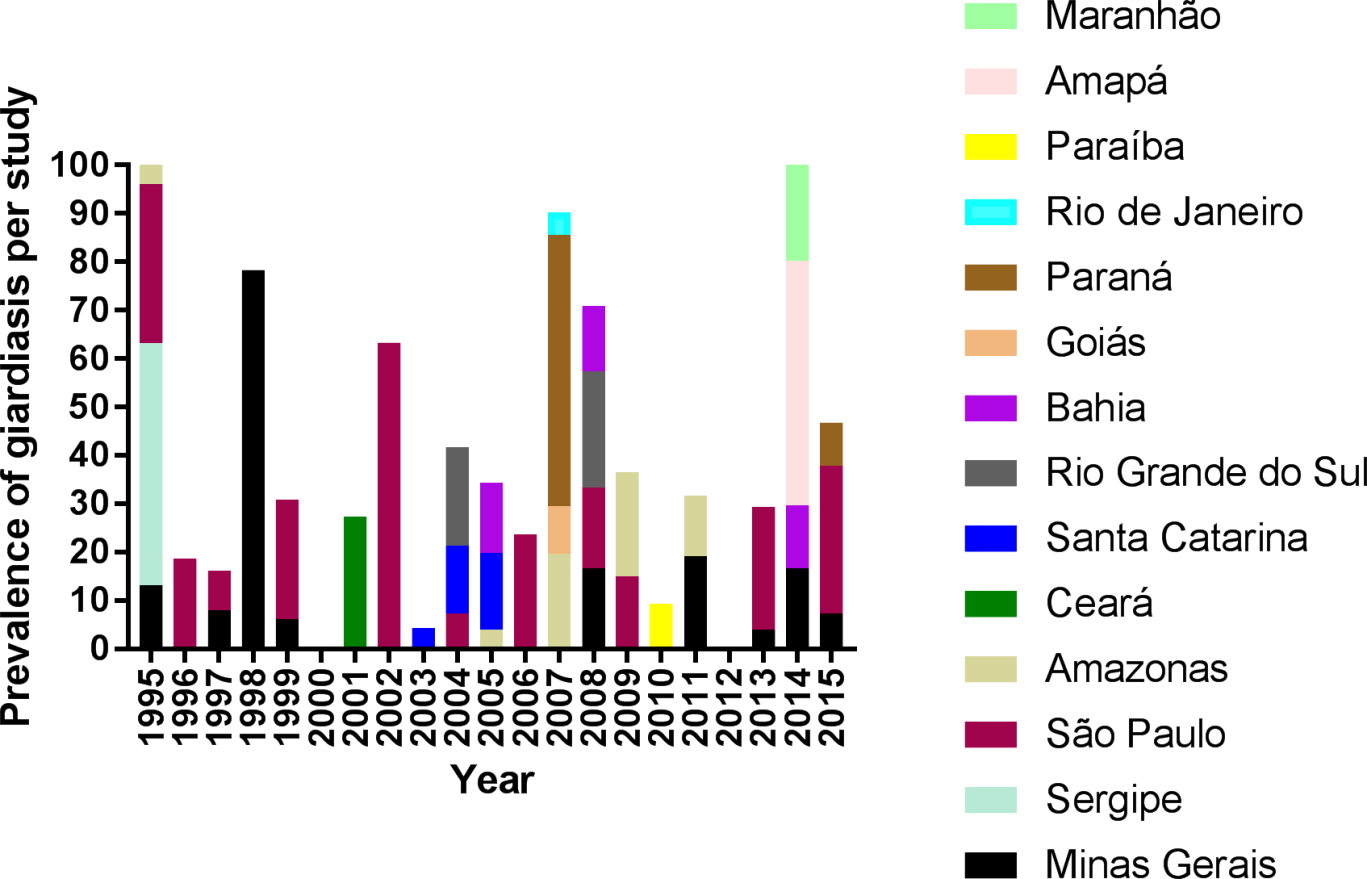
Prevalence of giardiasis according to studies estimating the prevalence in Brazilian states. The data was generated from the S1 Table and is an attempt of showing the description of prevalence of giardiasis in humans for each study performed in some Brazilian states along the 20 years analyzed.

According to the studies selected for evaluation of prevalence, giardiasis is present in all five Brazilian regions: southeast, south, northeast, north and mid-west with findings of higher prevalence in the Southeast region, the most populous one **(Fig 3)**.

**Fig 4:**
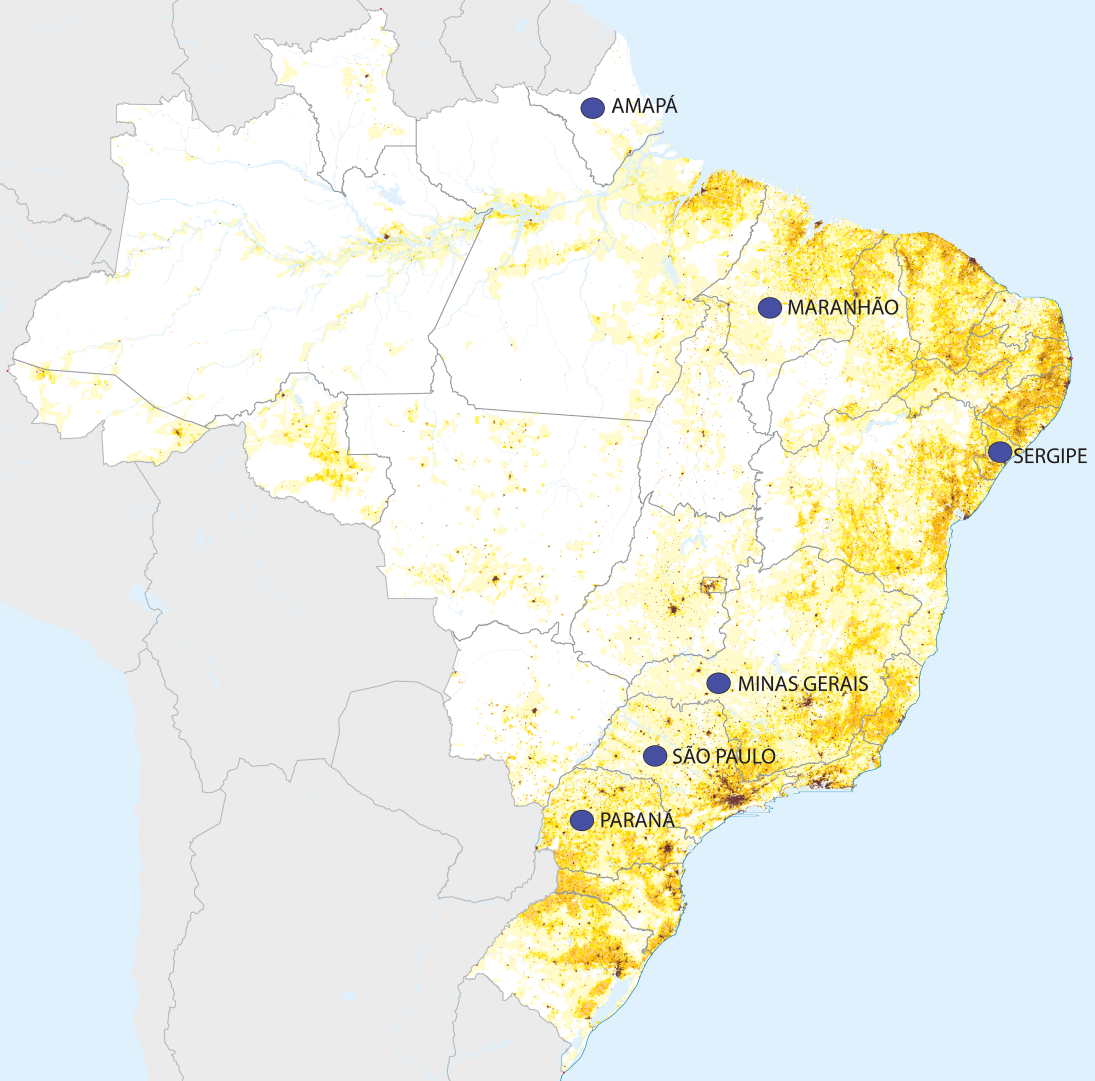
Brazilian states with more than 30% of prevalence of giardiasis in a population density map. Yellow areas in the map reflect population density. The blue circles show the states with more than 30% in prevalence of giardiasis: Amapá, Maranhão, Minas Gerais, Sergipe, São Paulo and Paraná. The information regarding to prevalence of giardiasis was obtained from studies identified at S1 Table and the Brazilian map was adapted from Brazilian Institute of Geography and Statistics (https://www.ibge.gov.br/) with population density data from Census obtained in 2010. North region comprises the following states: Acre, Amapá, Amazonas, Pará, Rondônia, Roraima and Tocantins. Northeast states are Alagoas, Bahia, Ceará, Maranhão, Paraíba, Piauí, Pernambuco, Rio Grande do Norte and Sergipe. Midwest region comprises: Goiás, Mato Grosso, Mato Grosso do Sul and Federal District. Southeast states are Minas Gerais, Rio de Janeiro, São Paulo and Espírito Santo. Finally, South region is formed by Paraná, Santa Catarina and Rio Grande do Sul state.

The five states with the greatest populations according to the Brazilian Institute of Geography and Statistics (IBGE) are São Paulo, Minas Gerais, Rio de Janeiro, Bahia and Rio Grande do Sul. Although Rio de Janeiro is among the most populated states, there is a lack of studies evaluating the prevalence of giardiasis in this state. Here, we selected for evaluation all the studies that measured the prevalence of giardiasis in Brazil, independently of whether they involved children or adults **(S1 Table)**. An evaluation of prevalence for each state or for the country during the analyzed period, was not possible due to differences between the diagnostic methods used and number of patients enrolled for each study. In addition, most of the studies were performed in the Southeast region and even in this region there is a lack of studies for some specific years or states, which makes any kind of temporal comparison difficult. However, in **Fig 3**, we can evaluate the prevalence for some studies. Maximum prevalence reached 78.3% in Minas Gerais state in 1998 and 69.6% in São Paulo state in 1998 **(Fig 3)**. The other states with high prevalence (greater than 30%) were Maranhão, Amapá, Sergipe and Paraná **(Fig 4)**. The first three are considered poor states, and the fourth one is the 6^th^ most populated state of the country. Although an absolute comparison cannot be performed due to the greater number of studies in the southeast compared to the northeast region, also due to the limitation in the number of patients enrolled in each study, and to differences in the diagnostic methodologies, we suggest here that the prevalence of giardiasis is higher in the poorest or in the most populous states. We also analyzed the distribution of the genotypes per state, and the genes used to identify them **(Table 4)**.

**Table 4:** Detection of *G*. *duodenalis* assemblages in human hosts across Brazil according to molecular studies performed using the *gdh*, *tpi*, *bg* and *18S* parasite genes. **-** Methods used for each analysis include: 1- Sequence analysis of fragments; 2- Single-vessel multiplex real-time PCR (qPCR); 3- Restriction fragment length polymorphisms and DNA sequencing; 4- Allele-specific PCR; 5- Sequencing, Phylogenetic reconstruction and analysis of genealogical relationships.

Assemblages identified	Brazilian state	Genes investigated	Genotyping approach	Authors
AII (78.4%) B (21.6%)	São Paulo	glutamate dehydrogenase (gdh)	1	[39] 2007
A (15%) B (74%) Mixed (10.3%)	Ceará	18S rRNA	2	[74] 2008
AI (69.5%) AII(30.5%)	São Paulo	beta-giardin	1	[75] 2011
AI (96.7%) AII(3,3%)	São Paulo	beta-giardin	3	[38] 2007
B (100%)	Minas Gerais	glutamate dehydrogenase (gdh)	1	[76] 2012
(AI) single sample study	Minas Gerais	rRNA	4	[49] 2011
AI (16.6%) AIII (83.4%)	São Paulo	glutamate dehydrogenase (gdh) and triosephosphate isomerase (tpi)	3	[77] 2011
For HSP: A (100%) For β-giardin: A (53.3%) and B (46.7%)	Paraná	heat shock protein [HSP] and beta-giardin	3	[78] 2014
A(45%) B(46%) C(9%)	São Paulo	beta-giardin, glutamate dehydrogenase (gdh) and triosephosphate isomerase (tpi)	5	[31] 2014
AI (5.2%) AII (47.3%) BIV (47.3%	São Paulo	beta-giardin, glutamate dehydrogenase (gdh) and triosephosphate isomerase (tpi)	1	[34] 2015
AI (100%)	São Paulo	beta-giardin	3	[79] 2013
AI (3.7%) BIII (3.7%), AII (22.2%) BIV (70.4%)	Paraná	beta-giardin and glutamate dehydrogenase (gdh)	3	[47] 2015

**Fig 5:**
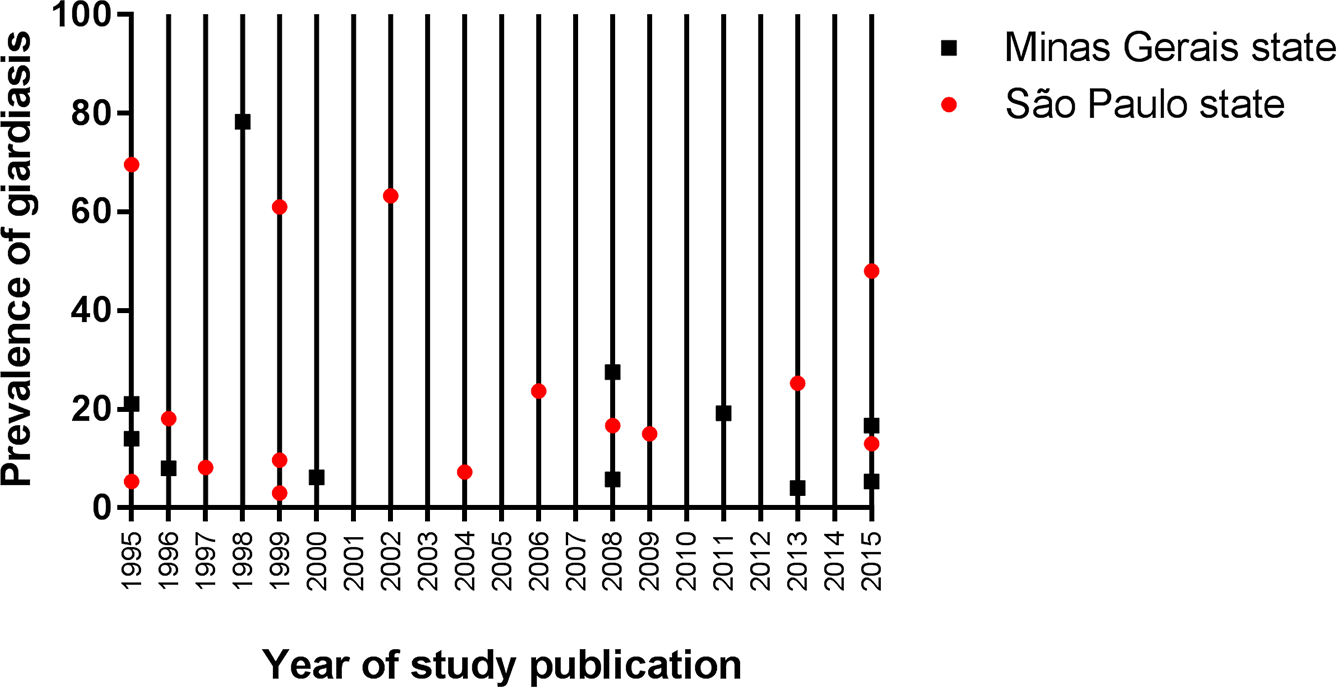
Publications determining the prevalence of giardiasis in the two most populated states of Brazil. Minas Gerais and São Paulo also presented the highest prevalence of giardiasis according to Fig 4. This graph shows how the prevalence of the disease has evolved over the years according to the published studies. Lines with two or more markers (red circles for São Paulo or black squares for Minas Gerais) represent more than one study per year.

Using data from the states with the highest prevalence, Minas Gerais and São Paulo, we performed a timeline comparison for the articles published between 1995 and 2015 in these states, although there was a limited number of publications for some years and although the same cities were not compared every year **(Fig 5)**.

### Giardiasis in Brazilian indigenous population

Brazil has a large indigenous population. More than 240 indigenous communities were counted in the last Brazilian census. These communities correspond to 896,917 people (0.47% of the Brazilian population) and 572,083 of these live in rural villages [10].

Some ethnicities of the indigenous population maintain their cultural heritage, and consequently, their dietary habits have not been totally changed by the urbanization process. In addition, although specific public policies for assistance to this population were established in Brazil, they appear to be insufficient for the needs of those people [80]. Some indigenous populations do not have access to treated water or bathrooms, and when they do exist they are usually shared among many people in the village. Most of the houses do not have solid floors or outdoor paving and the water is typically stored in uncovered buckets. This scenario is highly favorable to increased transmission of giardiasis.

According to Assis and colleagues, giardiasis was the second most prevalent disease among the Maxakali ethnicity [81]. They used a diagnostic method named TF-Test (Three Fecal Test). In this experiment, samples were collected on three alternate days in independent tubes, and then pooled for a double filtering by centrifugation. Among the 112 children studied, with ages ranging from one to 5 years old, 43 of them (38.4%) presented infection by *G*. *duodenalis*. Interestingly, the group that showed highest prevalence of giardiasis was the 40 to 49 years old adults. From the 23 samples collected from this group, 11 of them (47.8%) were positive for *G*. *duodenalis*. Forty-six percent of the population presented polyparasitism, defined by the presence of one or more intestinal parasites in the same host.

The Amazon region is where we find the greatest numbers of indigenous populations. In the northwestern part of the Amazon region, close to the Negro River, 90% of the population is indigenous. Boia and colleagues evaluated the prevalence of tuberculosis and intestinal parasitosis among the indigenous population in the Amazon region. They collected 313 samples from 54 houses (from villages within a main municipal area), and using the Coprotest method, they found 26 samples positive for *G*. *duodenalis* [82]. Another indigenous Amazon community was investigated for *G*. *duodenalis* prevalence by Hoffman method and direct examination techniques (performed on site, in the small village). They found the rate of giardiasis in the adults and children (0–15 years old) ranging from 44.8 to 52.9% [83]. The highest prevalence rate was found in children from 10 to 15 years old. In this community 80% of the samples were positive for at least one intestinal parasite. To evaluate whether public health policy was effective for this population, the study was repeated in the same community after 3 years. They found a diminished prevalence for *G*. *duodenalis*, but in general, the number of parasitic infections was not lower than it was three years before, demonstrating that those policies should be improved.

Indigenous communities utilize water from the river for many purposes, such as: cooking, washing vegetables, bathing and swimming [84]. Nishi et al. evaluated the prevalence of protozoa in the spring water, river water and treated water that serves an indigenous community in the south of Brazil [26]. *G*. *duodenalis* was the most prevalent protozoan and it was found in the river and treated water at rates of six cysts/L and two cysts/L, respectively.

Mato Grosso do Sul state is one of the less urbanized states in Brazil and hosts many indigenous populations. One of these communities presented a high rate of polyparasitism involving *G*. *duodenalis* [85]. The co-infection cases with *Giardia* in this community were with the following parasites: *Ascaris lumbricoides*, *Trichuris trichiura*, *Entamoeba coli*, *Entamoeba histolytica* and *Endolimax nana*. North of Mato Grosso do Sul state is Mato Grosso state that hosts a large Indian Reservation called Xingu, comprising the following tribes: Pavuru, Moygu, Tuiarare, Diauarum, Capivara and Ngojwere. In this community, Escobar-Pardo et al. evaluated the correlation of gastrointestinal infection by *H*. *pylori* with some intestinal parasites through multivariate analysis; *Giardia* was closely associated with *H*. *pylori* [86], as has been seen in other studies [87, 88].

**Table 5**shows the prevalence of giardiasis in each Brazilian indigenous tribe, as well as the number of individuals enrolled in each study.

**Table 5:** Prevalence of giardiasis found in indigenous population in Brazil. The table shows the indigenous tribe, the localization of the tribe across the country, prevalence, number of individuals enrolled in each study and the year of publication.

Indigenous tribe	Brazilian state	Prevalence of *Giardia* in fecal samples	Number of individuals in the study	Reference
*Maxakali*	Minas Gerais	43.7% (adults)	23	[81] 2013
*Tariana and Baniwa*, *belonging* *to the Arawak linguistic trunk;* *Tukano*, *Desana*, *Kubeo*, *Tuyuca*, *Pira-tapuya*, *Arapaso and Wanana*, *of the Eastern Tukano group;* *Hüpda*, *of the Maku* *Linguistic family*	Amazonas	10.7% (adults)	313	[82] 2009
*Parakaña*	Paraná	44.8%(1992)/21.2%(1995) (adults) 50% (1992)/ 13.5%(1995) (children)	22/30 58/80	[83] 1998
*Terena*	Mato Grosso do Sul	23% (children and adults)	134	[85] 2014
*Xukuru-Kariri*	Minas Gerais	16.6% (children and adults)	60	[89] 2015
*Mbyá- Guarani*	Rio Grande do Sul	28.6% (children)/ 15% (adults)	42/20	[90] 2012

### Transmission networks among *Giardia* hosts in Brazil

During the analysis of transmission networks using the three genes, *gdh*, *tpi* and *bg*, we were looking for the main reservoirs of the parasite. Those analyses took into consideration the number of times that host shifts occurred within phylogenetic trees constructed for three different genes. This analysis used prior information from phylogenetic analysis that determine the relationships among the sequences.

**Fig 6:**
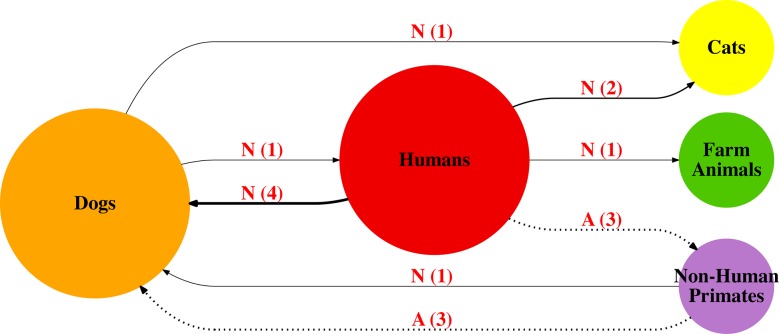
*Giardia duodenalis* transmission network based on sequences available for partial *beta-giardin* (n = 144). Solid Line/N = Non-Ambiguous Changes. Dotted line/A = Ambiguous Changes. Bolder solid lines indicate relatively more host shifts within route on the transmission graph. Number in parentheses represents number of shifts within tree from one host to another.

**Fig 7:**
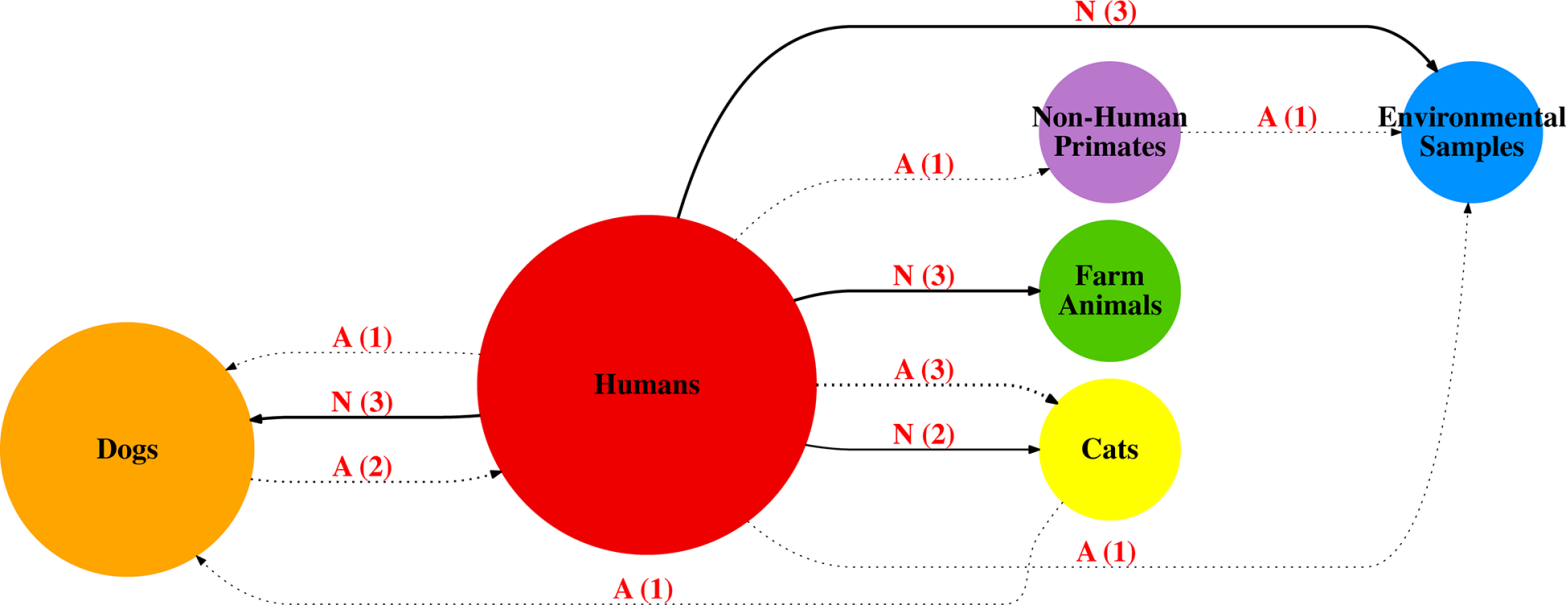
*Giardia duodenalis* transmission network based on sequences available for partial *tpi* (n = 148). Solid Line/N = Non-Ambiguous Changes. Dotted line/A = Ambiguous Changes. Bolder solid lines indicate relatively more host shifts within route on the transmission graph. Number in parentheses represents number of shifts within tree from one host to another.

**Fig 8:**
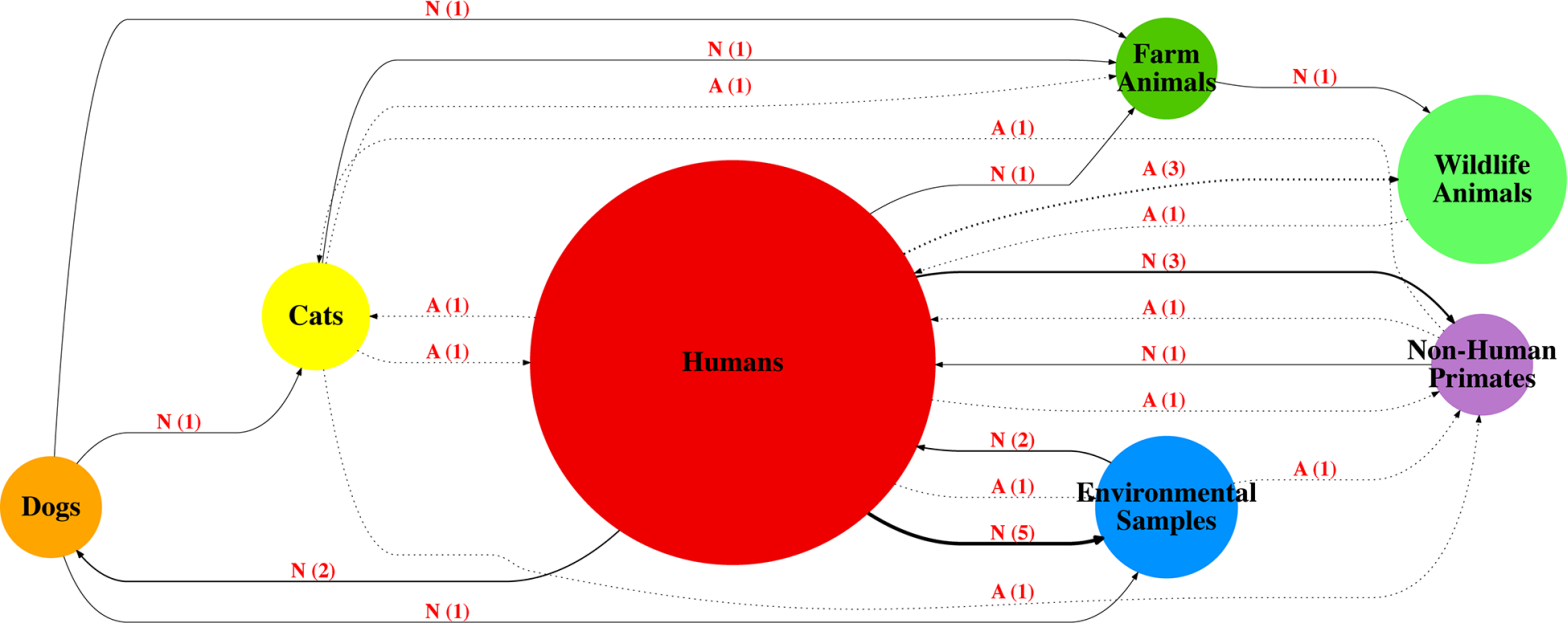
*Giardia duodenalis* transmission network based on sequences available for partial *gdh* (n = 342). Solid Line/N = Non-Ambiguous Changes. Dotted line/A = Ambiguous Changes. Bolder solid lines indicate relatively more host shifts within route on the transmission graph. Number in parentheses represents number of shifts within tree from one host to another.

The source of transmission (major centrality, determined by size of circle on figures), can be observed from the “Humans” category in Figs 6, 7 and 8. Also, “Humans” and “Dogs” share greater centrality on the *bg and tpi* transmission networks **(Figs 6 and 7)**, with multiple changes of host between each other. This is also observed for the *gdh* network **(Fig 8)**, although “Dogs” don’t have a great role compared to “Humans” on this network. On both *gdh* and *tpi* networks, an interaction between Humans and Environmental Samples (i.e. Stream Water, Sewage) can be observed with multiple changes from and between “Humans” and “Environmental Samples”. On the transmission networks “Cats”, “Farm Animals” and “Non-Human Primates” have a small interaction with “Humans”, with “Humans” mainly appearing as the source of transmission.

These analyses were performed independently on the three genes, but only represent the relationships given the current data available for Brazil compiled from different sources.

## Discussion

Although *Giardia* is considered the most commonly identified parasitic protozoan in human and animal feces in many parts of the world, including Brazil, giardiasis remains a neglected disease [3, 91]. Recently, the detection of *Giardia* spp. cysts has shown an increase in Brazil, especially among different water sources [16, 32, 33, 35]. However, studies reporting its detection are mostly concentrated in the South and Southeast regions of the country.

Data from the Ministry of Cities (National System of Information on Sanitation–SNIS, 2015) show that 82.5% of the Brazilian population was supplied with treated water in 2013, but more than 35 million people did not have this service. Collection of sewage was provided to 48.6% of the population; but it was not available for almost 100 million Brazilians. Only 39% of municipalities collect and treat 100% of their sewage. The most critical situations remain in the Northeastern and Northern regions. The Southeast is the region which provides the most sanitation services, i.e., sewage and water treatment, in the country. Thus, improvements will continue to be very slow if we anticipate universal provision of treated water, and sewage collection and treatment in 20 years in Brazil, according to the National Basic Sanitation Plan (2014–2033). Unfortunately, this scenario leads to continued dissemination of waterborne diseases, such as giardiasis, and we highlight the need for future investigations into better strategies for the control and prevention of this disease guided by molecular epidemiological studies. As Brazil is a country of continental dimensions, there are many regional differences in climatic conditions, cultural norms and social-economic development. In the most developed regions (Southeast and South), human developmental indicators and veterinary services available to pets can be comparable to those present in developed countries [41]. In contrast, in the Middle West, North and Northeast regions, the infrastructure is like those found in low-income countries. It is noteworthy that, regarding the detection of *Giardia* in animals, most of studies were performed in canine samples, probably due to the high number of specimens throughout the country associated with the abundance of this animal in many families as pets.

Throughout the period of this systematic review, most of the studies were performed using optical microscopy, although since 2011 studies involving molecular typing have become more common and even outstripped the ones based on traditional methodologies. With these molecular approaches, many studies identified the presence of zoonotic *Giardia* genotypes in dogs, cattle, sheep, cats, wildlife animals and non-human primates.

Data obtained in this systematic review provide, for the first time, insights into the evolution of the diagnostic tools used for *Giardia* detection in animals. However, there remain undeniable regional contrasts associated with the presence of natural reservoirs of the parasite with zoonotic potential. Moreover, our analyses show that many challenges remain regarding the expanded use of molecular techniques (mainly for clinical purpose) as well as updated public policies to avoid the spread of the disease through the population.

The transmission network analyses based on *gdh*, *bg*, and *tpi* genes identified the genotypes of *Giardia* detected in the Human host as the main source of transmission, which corresponds to a major centrality in comparison to the other groups. This result can be a consequence of the high frequency of zoonotic sequences in the Human group because it presents mainly isolates from genetic assemblages A and B (including sub-assemblages). The Dog group also shares a greater centrality with the Human group, probably due to the high incidence of sequences from genetic assemblages A and B, in addition to the host-adapted sequences from assemblages C and D. The other groups like Farm Animals and Cats present mainly host-adapted sequences E and F, respectively, which can decrease their role as reservoirs of zoonotic isolates and consequently reduce their importance in the transmission network. The interaction between Humans and Environmental groups also provides evidence of the relationship between these sources, even though the Environmental group presents fewer sequences than the Human group, a profile also observed in the Non-human primates group.

Although more studies are needed to determine the major centrality of *Giardia* obtained from humans, this review provides information on the sources of transmission and interactions between groups in a long-term analysis. More data are also necessary to enable studies that can build meaningful transmission networks with species resolution instead of merely groups. Nevertheless, groups that presented a minor role in the transmission network can also contribute to the zoonotic transmission and should be included in future studies.

### Conclusion

This study is the first systematic review of giardiasis prevalence and epidemiology in humans, animals and water. It confirms that giardiasis should be addressed as a major concern in health policies for tropical disease reduction. Improvements in diagnosis, prevention and treatment are necessary tools for fighting this disease in Brazil.

## Supporting information

S1 Fig PRISMA Flow chart describing the strategy used for selecting studies for inclusion in the final analysis. The terms «Giardia*» and «Brazil» were searched in four databases: PubMed, Embase, Scopus and SciELO.
(DOC)

S2 Fig PRISMA Checklist. (DOC)

S3 Fig Phylogenetic tree resulting from maximum likelihood tree search under the GTRCAT model of substitution a data set based on *bg* partial gene coding region for 144 taxa (143 isolates of *Giardia duodenalis* and one *Giardia cati* as outgroup). The scale bar for the branch lengths is based on an estimate of number of substitutions on average per site. The table in the top left corner of the figure is a color based key for hosts for the different isolates.
(TIF)

S4 Fig Phylogenetic tree resulting from maximum likelihood tree search under the GTRCAT model of substitution a data set based on *gdh* partial gene coding region for 342 taxa (341 isolates of *Giardia duodenalis* and one *Giardia psittaci* as outgroup). The scale bar for the branch lengths is based on an estimate of number of substitutions on average per site. The table in the top left corner of the figure is a color based key for hosts for the different isolates.
(TIF)

S5 Fig Phylogenetic tree resulting from maximum likelihood tree search under the GTRCAT model of substitution a data set based on *tpi* partial gene coding region for 148 taxa (147 isolates of *Giardia duodenalis* and one *Giardia microti* as outgroup). The scale bar for the branch lengths is based on an estimate of number of substitutions on average per site. The table in the top left corner of the figure is a color based key for hosts for the different isolates.
(TIF)

S1 Table Distribution of studies evaluating the prevalence of giardiasis in Brazil. The information is presented chronologically, beginning with the older studies.
(DOCX)
