# Supplementary material for: Giardiasis as a neglected disease in Brazil: Systematic review of 20 years of publications
Source: PLoS Negl Trop Dis. 2017 Oct 24;11(10):e0006005. doi: 10.1371/journal.pntd.0006005 (PMC5678545; doi:10.1371/journal.pntd.0006005)
Supplement: S1 Fig — The terms «Giardia*» and «Brazil» were searched in four databases: PubMed, Embase, Scopus and SciELO. (DOC) [file pntd.0006005.s001.doc]

**Screening**

**Included**

**Eligibility**

**Identification**

Records identified through databases searching
(n = 1049)

Additional records identified through other sources
(n = 2)

Records after duplicates removed
(n = 987)

Records screened
(n = 763)

Records excluded
(n = 520)

Full-text articles assessed for eligibility
(n = 243)

Full-text articles excluded
No data on prevalence = 4

No data on genotyping = 2

Review articles = 5
Related to sewage, not water = 3

No full text = 4

(n = 19 )

Studies included in qualitative synthesis
(n = 224 )

Studies included in quantitative synthesis (meta-analysis)
(n = N/A)
