# Supplementary figures and images for: Giardiasis as a neglected disease in Brazil: Systematic review of 20 years of publications
Source: PLoS Negl Trop Dis. 2017 Oct 24;11(10):e0006005. doi: 10.1371/journal.pntd.0006005 (PMC5678545; doi:10.1371/journal.pntd.0006005)

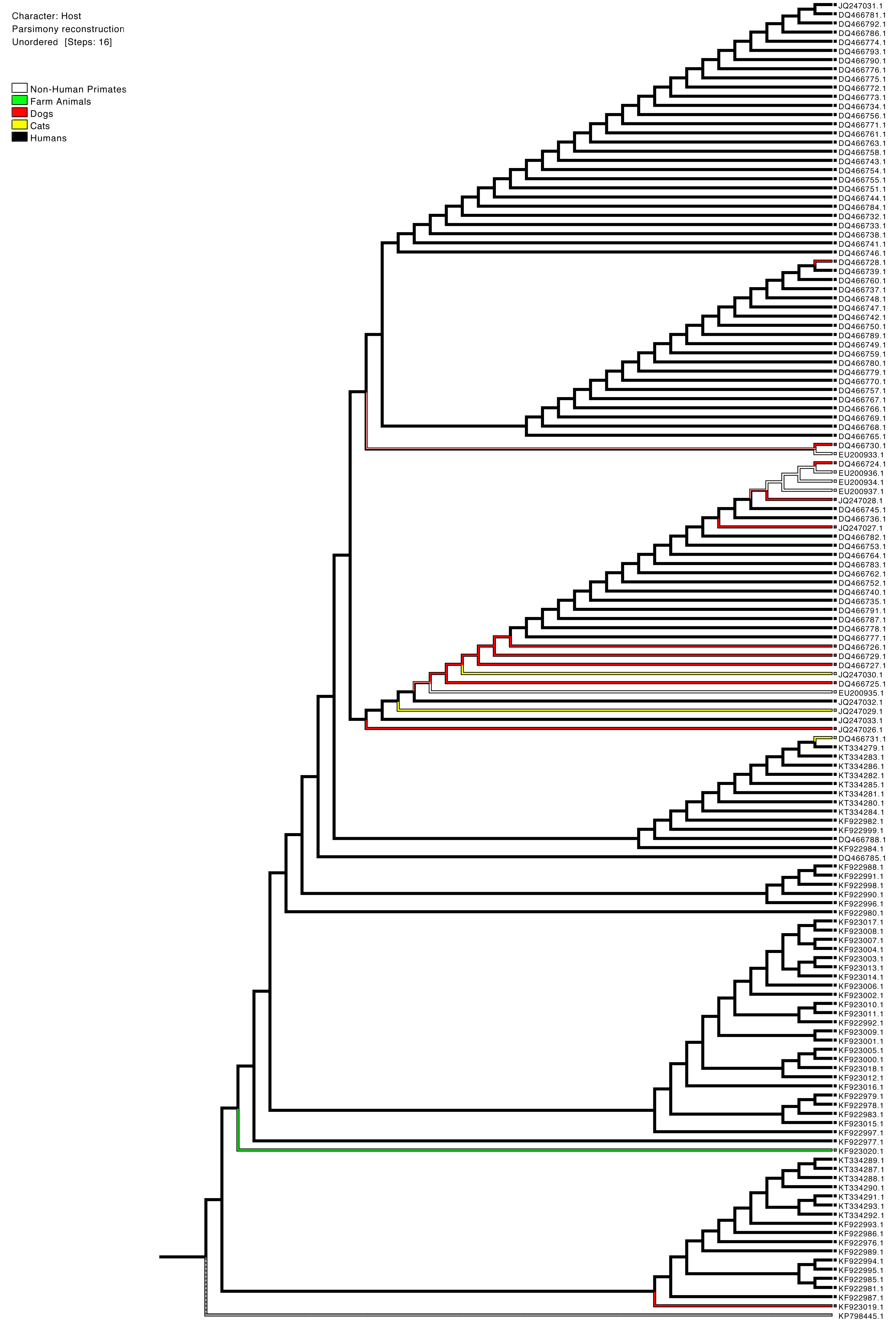

Supplement: S3 Fig — The scale bar for the branch lengths is based on an estimate of number of substitutions on average per site. The table in the top left corner of the figure is a color based key for hosts for the different isolates. (TIF) [file pntd.0006005.s003.tif]

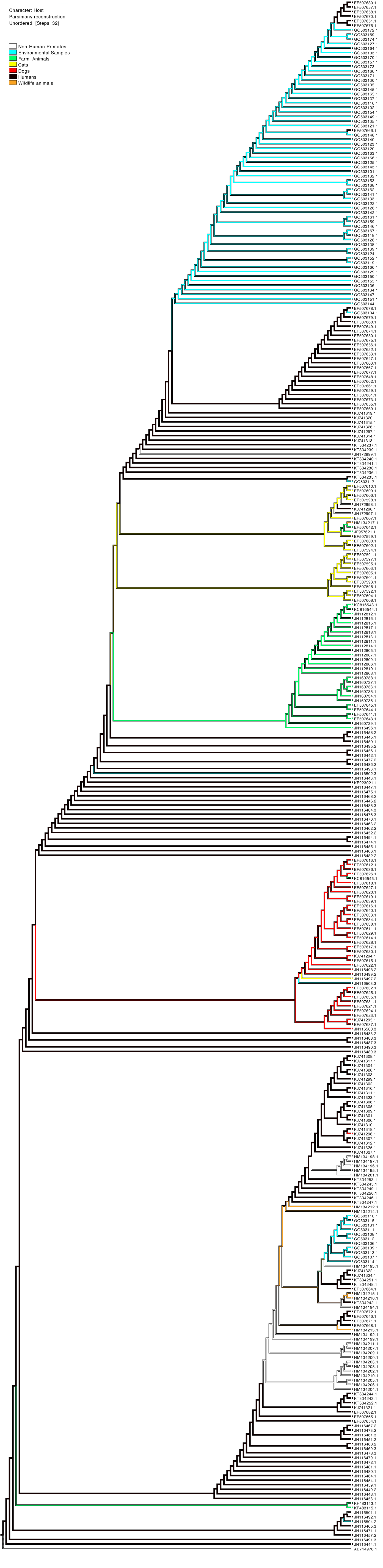

Supplement: S4 Fig — The scale bar for the branch lengths is based on an estimate of number of substitutions on average per site. The table in the top left corner of the figure is a color based key for hosts for the different isolates. (TIF) [file pntd.0006005.s004.tif]

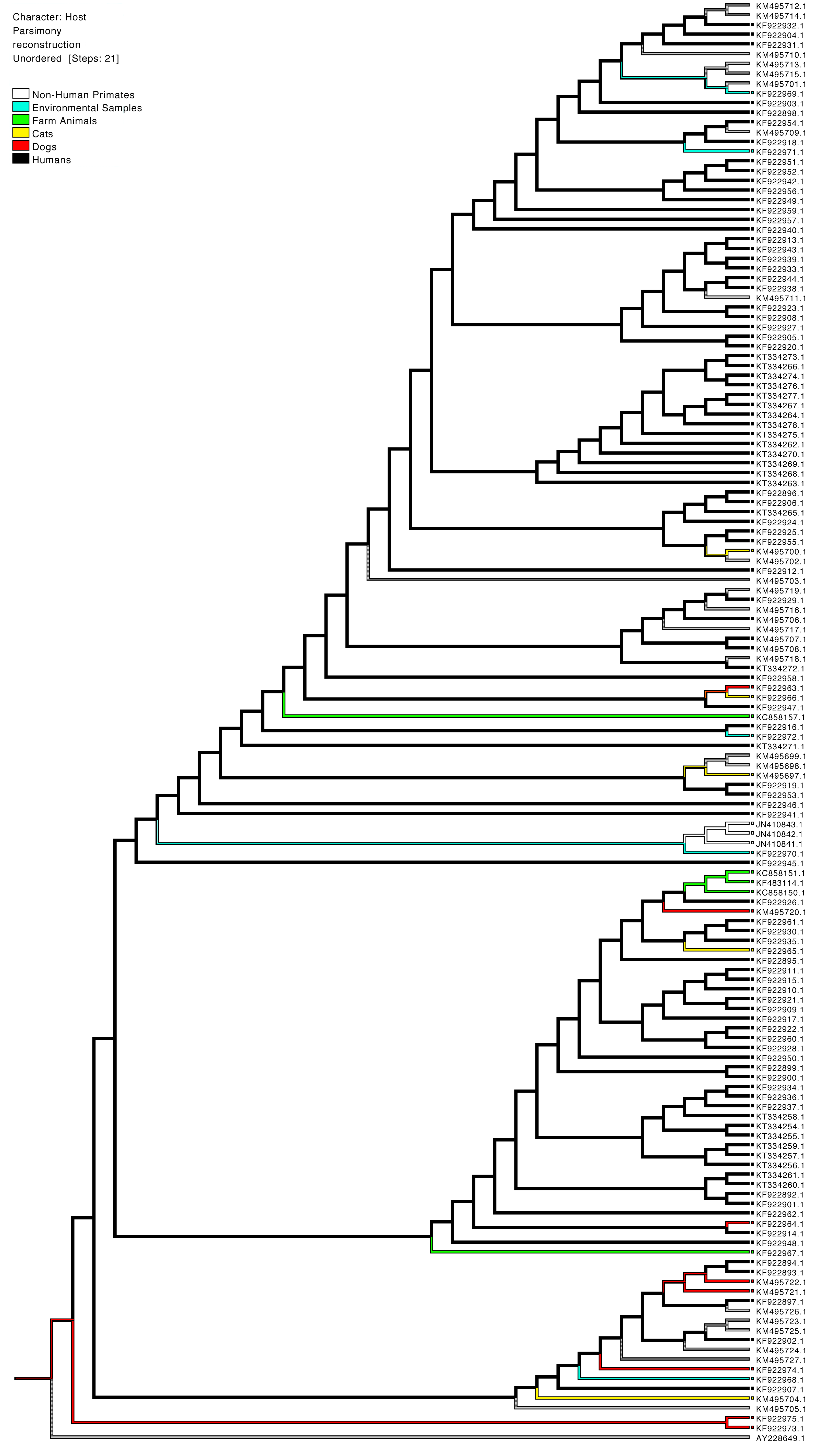

Supplement: S5 Fig — The scale bar for the branch lengths is based on an estimate of number of substitutions on average per site. The table in the top left corner of the figure is a color based key for hosts for the different isolates. (TIF) [file pntd.0006005.s005.tif]
