# Supplementary material for: Giardiasis as a neglected disease in Brazil: Systematic review of 20 years of publications
Source: PLoS Negl Trop Dis. 2017 Oct 24;11(10):e0006005. doi: 10.1371/journal.pntd.0006005 (PMC5678545; doi:10.1371/journal.pntd.0006005)
Supplement: S1 Table — The information is presented chronologically, beginning with the older studies. (DOCX) [file pntd.0006005.s006.docx]

**S1 Table - Distribution of studies evaluating the prevalence of giardiasis in Brazil.** The information is presented chronologically, beginning with the older studies.

| **Prevalence** | **Brazilian state** | **Study (year)** |
| --- | --- | --- |
| 50% | Sergipe | [75] 1995 |
| 5.36% | Minas Gerais | [76] 1995 |
| 21.1% | Minas Gerais | [77] 1995 |
| 10.4% | São Paulo | [78] 1995 |
| 69.6% | São Paulo | [79] 1995 |
| 18.6% | Sao Paulo | [80] 1996 |
| 8% | Minas Gerais | [81] 1997 |
| 8.2% | São Paulo | [82]1997 |
| 78.3% | Minas Gerais | [83] 1998 |
| 17.4% | Amazonas | [84] 1999 |
| 3% | São Paulo | [85] 1999 |
| 61.1% (public school)  9.7% (private school) | São Paulo | [86] 1999 |
| 6.2% | Minas Gerais | [87] 1999 |
| 27.4% | Ceará | [88] 2001 |
| 63.3% | São Paulo | [89] 2002 |
| 4.3% | Santa Catarina | [90] 2003 |
| 20.3% | Rio Grande do Sul | [91] 2004 |
| 14% | Santa Catarina | [92] 2004 |
| 7.3% | São Paulo | [93] 2004 |
| 1% | Amazonas | [94] 2005 |
| 15.9% | Santa Catarina | [95] 2005 |
| 7% | Amazonas | [96] 2005 |
| 14.5% | Bahia | [97] 2005 |
| 23.7% | São Paulo | [98] 2006 |
| 19.6% | Amazonas | [99] 2007 |
| 9.9% | Goiás | [100] 2007 |
| 56% | Paraná | [101] 2007 |
| 4.7% | Rio de Janeiro | [102] 2007 |
| 16.7% | São Paulo | [103] 2008 |
| 24% | Rio Grande do Sul | [104] 2008 |
| 27.5% | Minas Gerais | [105] 2008 |
| 5.8% | Minas Gerais | [106] 2008 |
| 13.5% | Bahia | [107] 2008 |
| 21.5% | Amazonas | [108] 2009 |
| 15% | São Paulo | [93] 2009 |
| 9.3% | Paraíba | [109] 2010 |
| 19.2% | Minas Gerais | [110] 2011 |
| 12.5% | Amazonas | [111] 2011 |
| 4% | Minas Gerais | [112] 2013 |
| 25.3% | São Paulo | [113] 2013 |
| 12.9% | Bahia | [114] 2014 |
| 50.6% | Amapá | [115] 2014 |
| 50% | Maranhão | [115] 2014 |
| 16.7% | Minas Gerais | [116] 2014 |
| 13% (mean from two cities) | São Paulo | [34] 2015 |
| 48% | São Paulo | [117] 2015 |
| 8.9% | Paraná | [48] 2015 |
| 7.4% | Minas Gerais | [118] 2015 |
